# Some Thoughts Concerning Old Remedies, Etc.

**Published:** 1883-12-20

**Authors:** Harvey L. Byrd

**Affiliations:** President and Professor of Obstetrics and Diseases of Women and Children in the Baltimore Medical College, Baltimore, Maryland


### SOME THOUGHTS CONCERNING OLD REMEDIES NOW CONSIDERED ALMOST OBSOLETE BY PHYSICIANS—TARTAR EMETIC, FOR EXAMPLE.

By HARVEY L. BYRD, M. D.,

President and Professor of Obstetrics and Diseases of Women and Children in the Baltimore Medical College, Baltimore, Maryland.

Whilst the present age may be considered generally as a progressive one, and in a great many respects is really such in fact, as may be seen in the numerous accessions that have been made in the various arts, and in many departments of science likewise, which are seemingly permanent additions to what was known before ; and, therefore, calculated to benefit mankind in various ways, yet so far as it relates to the medical profession it cannot be properly regarded as a utilitarian one, certainly not in the broad acceptation in which some have thought proper to apply that term to the advancements taking place in the latter half of the nineteenth century. Hence we pause to consider that it is lacking in conservatism, in our calling at least, in a conspicuous degree.

The adaptation of means to ends that so generally marks discoveries as they are utilized from day to day at the present time, in a manner and to a degree probably never equalled before in the various arts and sciences, including medicine, would seem to indicate that an attempt like this to revive an old remedy and bring it prominently before the profession would be truly "a work of supererogation."

But when the thoughtful mind reverts to the great benefits it has seen result from tartar emetic, and contemplates and compares the action of the remedies that have been substituted for it and the results obtained, there will be found sufficient reason to "give us pause," and to ascertain whether our great zeal in behalf of *new remedies* is not causing us to drift away from that which is good to that or those remedies which are no better at least than it is, and whether or not the tendency of the profession is to ignore many other old remedies and useful experiences of past ages, and press them to the rear, where they have not been actually forgotten, when making plans for new discoveries or new facts in the healing art.

Again, it may be observed of a few modern remedies even, or those of comparatively recent introduction, that the tendency in some instances is to permit them to fall still-born ere sufficient time is given for their proper development or utilization, because unsupported by the sanction of a great name, in order, seemingly, to afford larger space for others that appear to offer more brilliant prospects of usefulness to the profession or a wider fame to the discoverer.

Whilst always ready to remove obstructions and to facilitate progress and discovery by all proper means I often think that more enduring and substantial results would be certainly reached if we could delay just long enough to "prove all things and to hold fast only to that which is good" in medicine, as is done in almost all the other departments of human affairs.

I am emboldened to step to the front in the advocacy of tartar emetic, from seeing the good effects upon the profession that followed an article I had the temerity to publish in the Medical and Surgical Reporter of Philadelphia, in 1872, entitled Blood-Letting in Disease.

I am thoroughly satisfied, after four decades of experience as a physician engaged in active professional work, that, next to blood-letting, the tartrate of antimony and potash is absolutely without a peer or rival as an antiphlogistic agent in our therapeutic resources, and that it may, in some cases, be substituted for blood-letting, even, without detriment, when certain circumstances or conditions do not absolutely demand the use of that old and peerless remedy in inflammation.

I am conscious of the import of the language I am using, and desire that I may not be misunderstood in regard to it. And I wish to add, still further, that, like blood-letting, the necessity for its use in practice is now as great as it ever was at any time in the history of the article. After venesection, in acute inflammatory affections, I have found it produce its most strikingly marked beneficial effects, and feel fully warranted in saying that the most skeptical member of the profession would not doubt its wondrous power for good could its action be observed in a single case. But, as already stated above, its field of usefulness covers absolutely all cases of febrile and inflammatory affections that are unattended with inflammation or considerable irritation of the gastric mucous membrane. Those conditions only contra-indicate its internal employment in any form of disease whatsoever, or in any pathological condition attended with a full or even moderately tense and quick pulse, with dry skin and paucity of the secretions generally. It will be seen from these statements that, with the single exception of calomel, it is capable of doing good in a larger number of diseases than any other remedy in the hands of the medical practitioner. With these remarks I might conclude this paper, and, were I not aware of the fact that there are a large number of practitioners who have never used the article at all, would probably be inclined to do so. But for the use of such, and of those who have permitted other and more recent articles to monopolize its place in their therapeutic resources, I feel that the interests of science demand that a few more words should be added regarding its mode of administration, etc.

In doses of from one-eighth to one-tenth of a grain, alone or in conjunction with opium or one of its salts or preparations, I expect good results from it when given as an antiphlogistic or antipyretic, expectorant, diaphoretic, diuretic, or as an alterative. I never prescribe it as an emetic, unless no other article of that class is convenient, and am not prepared to speak of its *tolerance*, as mentioned by Rasori many years ago, in acute diseases from personal experience. Thus, I find it a valuable agent in most forms of fever, in bronchitis, in pneumonia, in croup and laryngitis, in torpid conditions of the liver, in certain chronic cutaneous diseases, and in sick headache, etc. It is as valuable in lessening the force and frequency of the circulation as veratrum or aconite, and, being tasteless in the proper dose, is almost absolutely free from disagreeable or unpleasant effects, and thus is generally preferable to either of them.

The foregoing strong commendation of tartar emetic in this paper will be endorsed, I feel quite sure, by those practitioners who would preserve the old landmarks in our therapeutics, and are unwilling to drift too far away from the moorings of well-tried experience, merely to follow fashion or for the sake of novelty in practice. And if it should prove the means of adding a most valuable and trustworthy article to the therapeutic repertory of a physician unaccustomed to or inexperienced in its use in the treatment of his patients, another most important object will have resulted from its preparation and its publication in the Medical Times.—*Medical Times.*

